# Fast Hydrogen Sorption Kinetics in Mg-VCl_3_ Produced by Cryogenic Ball-Milling

**DOI:** 10.3390/ma16062526

**Published:** 2023-03-22

**Authors:** Karina Suárez-Alcántara, Nadia Isabel Flores-Jacobo, Mayara del Pilar Osorio-García, José Gerardo Cabañas-Moreno

**Affiliations:** 1 Morelia Unit of Materials Institute Research, National Autonomous University of Mexico, Antigua Carretera a Pátzcuaro No. 8701, Col. Ex Hacienda de San José de la Huerta, Morelia CP 58190, Mexico; 2 Nanoscience and Nanotechnology Program, Centro de Investigación y de Estudios Avanzados (CINVESTAV-IPN), Av. Instituto Politécnico Nacional 2508, San Pedro Zacatenco, Gustavo A. Madero, Ciudad de México CP 07360, Mexicojcabanasm@cinvestav.mx (J.G.C.-M.)

**Keywords:** hydrogen storage, magnesium, VCl_3_ additive, cryogenic ball milling, fast kinetics

## Abstract

Hydrogen storage in Mg/MgH_2_ materials is still an active research topic. In this work, a mixture of Mg-15wt.% VCl_3_ was produced by cryogenic ball milling and tested for hydrogen storage. Short milling time (1 h), liquid N_2_ cooling, and the use of VCl_3_ as an additive produced micro-flaked particles approximately 2.5–5.0 µm thick. The Mg-15wt.% VCl_3_ mixture demonstrated hydrogen uptake even at near room-temperature (50 °C). Mg-15wt.% VCl_3_ achieved ~5 wt.% hydrogen in 1 min at 300 °C/26 bar. The fast hydriding kinetics is attributed to a reduction of the activation energy of the hydriding reaction (E_a hydriding_ = 63.8 ± 5.6 kJ/mol). The dehydriding reaction occurred at high temperatures (300–350 °C) and 0.8–1 bar hydrogen pressure. The activation energy of the dehydriding reaction is 123.11 ± 0.6 kJ/mol. Cryomilling and VCl_3_ drastically improved the hydriding/dehydriding of Mg/MgH_2_.

## 1. Introduction

Hydrogen as an energy vector can be of interest [1,2] because it can be used in a wide range of energy generators, from turbines to fuel cells [3]. Hydrogen is also an important industrial (chemical and petrochemical) reagent. Other applications include its use in heat storage systems [4]. However, all applications have in common the need for suitable hydrogen storage systems. Mg/MgH_2_ is an interesting hydrogen storage system due to the reversibility of hydriding/dehydriding reactions without extreme loss of storage capacity, relatively low cost of Mg, high hydrogen content, etc. [5,6]. Ideally, low temperature dehydriding of MgH_2_ must be achieved for many applications. However, the lower temperature of the dehydriding reaction is dictated by the thermodynamics of MgH_2_, i.e., by the MgH_2_ formation enthalpy [7,8]. Using additives or special processing techniques has proven to be of little help in modifying the thermodynamics of MgH_2_. Thus, MgH_2_ is restrained to high-temperature (300–350 °C) applications. Nowadays, the research on hydrogen storage using Mg/MgH_2_ systems is still of interest to surpass kinetic limitations [5,6].

MgH_2_ has a total hydrogen content of 7.6 wt.%; however, only in a few cases full-capacity release/storage is achieved. Reaction temperature, pressure and time, surface oxide conditions of Mg/MgH_2_ particles, additives, and sample processing such as ball milling tremendously influence the hydrogen uptake level and kinetics. To date, several materials have been tried as additives or catalysts using different processing techniques. The list of additives or catalysts includes but is not limited to oxides such as Nb_2_O_5_ [9,10], intermetallics such as Mg_2_Ni [11], metallic alloys such as NiMn_9.3_Al_4.0_Co_14.1_Fe_3.6_ [12], transition metals [10,13], transition metals halides [14,15,16], metallic nanoparticles such as Ni [17], sulfides [18], co-catalysts of different substances [11,14], and many other compounds. The role of the catalyst is to enhance the dissociation and recombination rate of hydrogen and to improve H diffusion as a “hydrogen pump” [19].

V-containing compounds are interesting catalysts because of their affinity towards electrons due to unoccupied *d* orbitals [20]. VF_4_ added to Mg_99_Ni was proposed to form VH_0.91_ and MgF_2_ during mechanical milling [15]. In that work, the VH_0.91_ was acting as a hydrogen pump [15]. Other V-containing compounds have improved the hydriding/dehydriding of Mg/MgH_2_ [15,21,22,23]. In particular, the addition of VCl_3_ [24,25,26] resulted in quick sorption kinetics [27,28,29]. A survey on the Web of Science on the topics Mg or MgH_2_, VCl_3_, and hydrogen storage produced only 12 published papers. Of them, three used VCl_3_ as a co-catalyst; and in the other three, the MgH_2_ was mixed with other hydrogen storage materials. Of the papers on MgH_2_-VCl_3_ [24,25,26,27,28,29], the information on kinetics or thermodynamics is incomplete, as we detail in the discussion section. In comparison, a survey in the same database of MgH_2_, Nb_2_O_5_, and hydrogen storage produced 272 papers. This indicates the need for more studies of VCl_3_ as an additive, exploring new preparation conditions aimed at better kinetic results.

On the other hand, the reported amount of VCl_3_ added to MgH_2_ spans from 5 wt.% to 23.9 wt.% (i.e., up to 5 mol%) [25,26,29,30,31]. In the present work, we explored the use of a relatively high amount of VCl_3_, but in between the amounts reported [25,26,29,30,31]: 15 wt.% (or 2.65 mol%). It represents a primary component in the mixture intended for extensive interaction with Mg and MgH_2_ while maintaining an acceptable theoretical hydrogen capacity (6.46 wt.%). The use of high amounts of additives is rather common in the hunt for improving hydrogen storage kinetics [32]. For example, 2 mol% of heavy substances such as NbF_5_ or Nb_2_O_5_ in MgH_2_ corresponds to 12.7 and 16.7 wt.%, respectively [10,33]. Additionally, we remark that the starting material in most of the referenced papers is MgH_2_. Here, we explored the use of Mg as the starting material because, in many countries such as ours, the commerce of MgH_2_ is forbidden.

The most popular processing techniques of hydrogen storage materials include but are not limited to mechanical milling, reactive milling, cold rolling, high-pressure torsion, etc. [5,34]. Cryomilling is not as popular as room-temperature milling. However, cryomilling of ductile materials such as Mg can have advantages such as the reduction of cold welding and quick reduction of particle size [34]. A survey on the Web of Science on the topics “cryomilling” and “hydrogen storage” reported 18 papers, seven of them related to Mg/MgH_2_ catalyzed or co-catalyzed with different materials [10,32,33,35,36,37,38], and in none of them, the catalyst is VCl_3_. Here, we used cryogenic ball milling to obtain a Mg-VCl_3_ mixture as a new approach to improve Mg kinetics for hydrogen storage.

## 2. Materials and Methods

### 2.1. Mixtures Preparation

High purity VCl_3_ (Aldrich, anhydrous, 99.998% purity), and Mg powder (Alfa-Aesar, −325 mesh, 99.86 purity), were used as received. Then, 0.85 g of Mg and 0.15 g of VCl_3_ were weighed and deposited in a cryomilling vial. Next, 1 g of the powder mixture was milled with 20 g of balls. Specifically, 6 yttria-stabilized zirconia balls of 1 cm diameter were used in a stainless-steel cryomilling vial of 50 mL volume. The cryomilling vial was closed under an argon atmosphere of a glove box and transferred to the cryogenic mill (Retsch^®^, Haan, Germany). Then, the milling vial was fixed inside a chamber of the mill. The liquid N_2_ circulates between the exterior of the milling vial and the interior of the chamber. The liquid N_2_ circulates both in pre-cooling mode and when the chamber is moving (milling). The cryogenic mill of Retsch^®^ is a fully automatized machine that allows programming cycles of pre-cooling, milling, and pauses with agitation rates between 5 and 40 Hz [39]. The cryomilling vial, loaded with Mg, VCl_3_ and the balls, was pre-cooled for 10 min in a liquid N_2_ flow with a movement of the vial of 5 Hz (300 rpm). Once reached −196 °C, the powders were milled in 6 cycles of 10 min at 25 Hz (1500 rpm) agitation rate, and 1 min at 5 Hz (300 rpm) agitation rate. During the milling process, a constant flow of liquid N_2_ cooled the milling vial. The cryomilled mixture of Mg and VCl_3_, hereafter Mg-15wt.% VCl_3_, was recovered and characterized both in as-milled and hydrided forms. All materials were handled and stored in a protective argon atmosphere inside a Vigor^®^-glove box (less than 5 ppm O_2_ and H_2_O). For comparison, pure Mg was cryomilled in the same conditions as the Mg-15wt.%VCl_3_ mixture. Characterization of cryogenically milled Mg was performed and presented in the next sections as needed. In addition, for comparison, a mixture of Mg-15wt.% VCl_3_ was milled in similar conditions of time, agitation rate, etc., but at room temperature (i.e., not cryogenic cooling). Hereafter that sample is named Mg-15wt.% VCl_3_-RT.

### 2.2. Hydriding and Dehydriding Reactions

Temperature-programmed hydriding (TPH), temperature-programmed dehydriding (TPD), and isothermal hydriding/dehydriding experiments were carried out in a Sievert’s type apparatus of our design and construction [40]. The apparatus combines the feature of double (twin) lines (sample and reference) to eliminate small thermal effects on the reservoir and sample-holder volumes, with a Δ*p* = Δ*p_sample_* − Δ*p_reference_* approach [40]. Hydriding and dehydriding reactions were worked in pairs, i.e., in a cycle. Then, 0.3–0.5 g of samples were transferred to/from the Sievert’s-type reactor without oxygen contact within a sample holder with a closing (isolation) valve. Then, the system was purged by successive cycles of evacuation and high-purity argon flushing. Calibration and operation details were performed as reported elsewhere [40]. In brief, the calibration was performed to know the total void volume of the sample holder. This is undertaken by expanding a high-purity argon aliquot from the reservoir (well-known volume) to the sample holder (unknown void volume) at a constant initial temperature. After data collection for calibration, the argon was evacuated.

For the reference materials Mg and Mg-15wt.% VCl_3_-RT, an activation process was needed before the first hydriding reaction. The activation was performed by heating from room temperature up to 350 °C, 5 °C/min, in a dynamic vacuum for 2 h. After that, the sample was cooled to room temperature and kept in a vacuum overnight until the next hydriding experiment.

For TPH, the initial hydrogen pressure was fixed in the reservoirs and sample holders. After that, the sample was heated to the test temperature with a heating ramp of 5 °C/min. The TPH experiments were performed by heating up to 350 °C/26 bar or 350 °C/12 bar. After hydriding, the sample holder was quickly cooled to room temperature. Then, the remaining hydrogen was released and the pressure of the next TPD experiment was fixed. TPD experiments were performed by heating up to 350 °C/0.8 bar hydrogen pressure. After dehydriding experiments, the complete release of hydrogen from the sample was forced by applying dynamic vacuum for 30 min at 350 °C. In this way, we ensured a completely hydrogen-free material in the next experiment. One data set (pressure of reference, pressure of sample, temperature of reservoirs, and temperature of the sample) was collected every 5 s and processed as reported elsewhere [40] to obtain hydrogen uptake/release in wt.%. Data were processed as indicated in [40], and the hydrogen was treated as a real gas.

Isothermal hydriding and dehydriding experiments were performed by first calibrating the apparatus. Then, for hydriding experiments, the sample was heated to the test temperature in the vacuum. After reaching a stable isothermal condition, the expansion of a calculated aliquot of hydrogen from the reservoir into the sample holder was performed. The hydriding experiments were performed at 50 °C, 100 °C, 150 °C, 200 °C, 250 °C, and 300 °C at 26 bar; or 320 °C/12 bar. After hydriding, the remaining hydrogen pressure was released up to the testing pressure, the sample holder valve was closed, and the sample was quickly heated to 350 °C (20 °C/min). Once 350 °C was reached, the sample holder valve was opened. Dehydriding experiments were performed at 350 °C/0.8 bar, 350 °C/0.15 bar, and 320 °C/1 bar. One data set was collected every 1 or 0.5 s during the isothermal hydriding/dehydriding reactions, i.e., it was adjusted accordingly to the observed kinetics. At the end of dehydriding, the complete release of hydrogen was forced by applying dynamic vacuum for 30 min at 350 °C. Thus, we ensure a completely hydrogen-free material in the next experiment.

Pressure-Composition Isotherms (PCI) were performed in an isorb-100 machine of Quantachrome. Measurements of the isorb-100 machine of Quantachrome are based on the difference in pressure between a reference and the sample holder. A sample of about 0.75 g of as-milled materials was transferred to the machine without air contact utilizing a sample holder with an isolation valve. The calibration for void volume was performed with ultrahigh purity helium. Once at stable isothermal conditions, hydriding reactions were performed by a progressive increase (in about 25 steps) of the hydrogen pressure from 0.01 to 20 bar. Dehydriding reactions were performed by a progressive decrease (in about 25 steps) of the hydrogen pressure from 20 to 0.01 bar. PCI curves must be performed in equilibrium conditions, this translates to long testing times for real-world experiments. The equilibrium condition was assumed when the sample pressure presented a variation smaller than 0.1 × 10^−3^ bar during 12 min, or a maximum time of 240 min duration for each step. Reaching the equilibrium directed the change to the next step. The rigorous equilibrium condition criteria directed the total time employed at each experiment, normally between 4 and 6 days per whole hydriding/dehydriding curve. The experiments were performed at 295 °C, 300 °C, and 315 °C. Each experiment was performed with fresh samples of the same batch.

The hydrogen used during experiments (TPH, TPD, isothermal, and PCI) was of chromatographic purity. All the reported pressures correspond to the absolute pressure scale, 0.8 bar being the average atmospheric pressure in our location.

### 2.3. Physicochemical Characterization of the Materials

After TPH, TPD, isothermal hydriding/dehydriding, or PCI experiments, the samples were cooled down to room temperature and the remaining pressure was released. Afterward, the samples were recovered and characterized.

Differential Scanning Calorimetry (DSC) experiments were performed using SETARAM^®^ SENSYS EVO equipment. Samples of about 5–10 mg of hydrided (at 350 °C, 26 bar, 4th hydriding step) Mg-15wt.% VCl_3_ were placed in alumina crucibles and introduced into the DSC apparatus. Next, 20 mL/min of ultra-high purity Argon was used as a carrier gas. Heating ramps of 1, 5, and 10 °C/min were used in the tests. DSC data were extracted with Calisto software V1. The DSC apparatus works under a protective argon atmosphere inside a Vigor^®^-glove box (less than 10 ppm O_2_ and H_2_O).

X-ray diffraction (XRD) was performed in a D2 Phaser diffractometer of Bruker with a Cu tube (K_α_ = 1.540598 Å). The powders were compacted in a dedicated sample holder and covered with Kapton^®^ tape for protection against ambient oxygen and moisture during XRD data collection. Crystalline phase identification was performed with the help of Match! Software V1 and crystallographic databases: Inorganic Crystal Structure Database-Karlsruhe (ICSD) and Crystallography Open Database (COD).

SEM images of selected samples were collected in a JSM-IT300 microscope. Samples were dispersed on carbon tape inside the argon glove box and transferred to the SEM chamber minimizing the air exposure by using a glove bag. SEM images were obtained with secondary and backscattered electrons with 20 kV of acceleration voltage. Elemental mapping was performed by an SDD X-MaxN EDS detector of Oxford Instruments attached to the microscope.

## 3. Results

### 3.1. Hydriding and Dehydriding Reactions of Cryogenically Milled Mg-15wt.% VCl_3_

**Figure 1:**
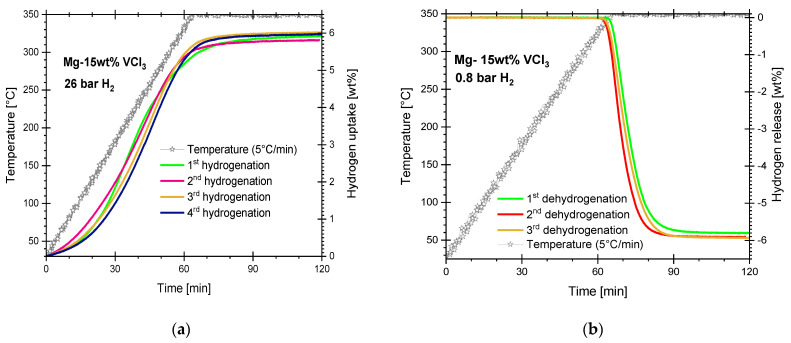
Hydriding/dehydriding cycles of cryogenically prepared Mg-15wt.% VCl_3_. (**a**) Hydriding at 350 °C and 26 bar. (**b**) Dehydriding at 350 °C and 0.8 bar. 5 °C/min heating rate.

Hydrogen storage in Mg typically requires an activation procedure to initiate the hydrogen uptake [41]. The activation process normally involves heating in a vacuum or in a hydrogen atmosphere. The activation process results in the fracture of particles, or at least, the formation of cracks that exposes non-oxidized surfaces and creates paths for faster hydrogen access to “clean” Mg surfaces. For the Mg-15wt.% VCl_3_ material produced with cryogenic ball-milling, the activation process was not necessary. The Mg-15wt.% VCl_3_ material started storing hydrogen as soon as it was exposed to hydrogen. The hydrogen uptake can be considered fast, starting almost from the beginning of the heating ramp and practically stopping after reaching 350 °C. The onset of hydriding is located at about 49–54 °C (Figure 1a). The on-set onset is defined in our research group at the point of reaching 0.1 wt.% hydrogen uptake or release in temperature-programmed hydriding/dehydriding experiments. Considering the 15 wt.% of VCl_3_ added to Mg, the maximum achievable hydrogen uptake by the formation of MgH_2_ is 6.46 wt.%; V-hydrides are not considered. The Mg-15wt.% VCl_3_ mixture reached a hydrogen uptake of 6 wt.% in the third and fourth hydriding steps. A complement to the activation process is hydriding/dehydriding cycling, normally a strong increase in kinetic or hydrogen storage levels is expected. However, as Figure 1 indicates, no changes in kinetic upon cycling of cryogenic Mg-15wt.% VCl_3_ was observed. Within the reproducibility of the test, a slight increase compared to the very first hydriding step can be noticed upon cycling. Dehydriding curves are presented in Figure 1b. Dehydriding of the cryomilled material Mg-15wt.% VCl_3_ occurred upon reaching 350 °C in this series of experiments. The dehydriding at 350 °C and 0.8 bar absolute pressure is completed in about 20 min after reaching the isothermal condition in TPD experiments.

**Figure 2:**
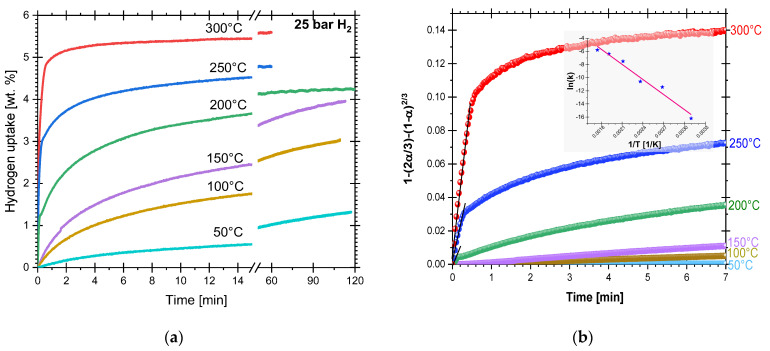
(**a**) Isothermal hydrogen uptake versus time. (**b**) Fitting to contracting volume, diffusion-controlled 3D growth with decreasing interface velocity model. Black lines indicate the fitting neighborhood (i.e., just a guide for the eye) for the 300, 250, and 200 °C curves. Inset of (**b**): Arrhenius plot of *ln*(*k*) versus the inverse of temperature for hydriding reactions of cryogenically prepared Mg-15wt.% VCl_3_.

Isothermal hydriding at 350 °C was impossible to follow without uncertainty because of its quickness, despite that the data collection ratio was increased up to 10 data sets each second. Just a few fractions of a second after a hydrogen aliquot was introduced at 350 °C, the reaction was running in full. From our experience with Mg-based hydrogen storage materials, hydrogen uptake in this cryogenically prepared Mg-VCl_3_ mixture is remarkably fast. Figure 2a presents the hydriding processes performed under isothermal (50–300 °C) conditions and 26 bar. For the isothermal curves, hydrogen uptake levels and kinetics improved with increasing temperature, being 5.6 wt.% hydrogen uptake after one hour at 300 °C, and 1.3 wt.% after two hours at 50 °C. Hydriding at 300, 250, and 200 °C completed 80% of their maximum hydrogen uptake in about 1, 3, and 14 min, respectively. For the tests at 150, 100, and 50 °C, the experiments were stopped after 2 h. In those conditions, the hydrogen uptake was 3.9, 3.0, and 1.3 wt.%, respectively. Hydriding kinetics were notably slower at those temperatures.

The kinetic models [42,43] known as surface reaction controlled (Equation (1)), Johnson–Mehl–Avrami (JMA, Equation (2)), and contracting volume (CV, Equations (3) and (4)), were used to fit the isothermal kinetics data, following the procedure in [42,43]:

(1) $$\alpha =\frac{wt(t)}{wt(max)}=kt,$$

(2) $${\left[-ln\left(1-\alpha \right)\right]}^{\frac{1}{n}}=kt,$$

(3) $${1-\left(1-\alpha \right)}^{\frac{1}{n}}=kt,\;$$

(4) $$1-\left(\frac{2\alpha }{3}\right)-{\left(1-\alpha \right)}^{\frac{2}{3}}=kt.\;$$

Equations (1)–(4) take the form *y* = *mx*, where *α* is the transformed fraction, *wt*(*max*) is the maximum hydrogen uptake or release or the maximum theoretical hydrogen content, *wt*(*t*) is the hydrogen uptake or release at a time *t*, *k* is the rate kinetic constant (obtained from the slope, *m*), *n* (*n* = 2 or 3) depends on the dimensionality of the growth of the new phase, and *t* is time. The three-dimensional diffusion-controlled growth model with decreasing interface velocity, Equation (4), produced the best fit for all hydriding reactions at different temperatures at the beginning of the reaction [42]. This model assumes that nucleation starts at the surface of Mg and that H-diffusion across the transformed phase (MgH_2_) is the rate-limiting mechanism [42]. However, as presented in Figure 2b, differences between low-temperature (50, 100, and 150 °C) and high-temperature (200, 250, and 300 °C) reactions were found. At any given temperature, a change in the kinetic mechanism with increasing reaction time was observed. The change is very evident in the high-temperature curves (Figure 2b), i.e., the model fits in the narrow time interval of the beginning of the reaction. For the high-temperature experiments, the fitting interval is indicated by black lines. For the low-temperature experiments, the fitting interval extends up to 30 min (not shown). At longer reaction times (>30 min), no model fitted the experimental data [42,44].

The application of the kinetics models (Equations (1)–(4)) unravels only part of the complexity of hydriding reactions; however, it is useful to extract values of the kinetic constant. Table 1 presents the calculated *k* and the time interval of the best fit of the model. The results in Table 1 corroborated the increasing kinetics with temperature. The inset of Figure 2b is the Arrhenius plot of *ln*(*k*) versus the inverse of temperature constructed with the estimated *k’s*. The activation energy of the hydriding reaction calculated from the slope is 63.8 ± 5.6 kJ/mol.

**Figure 3:**
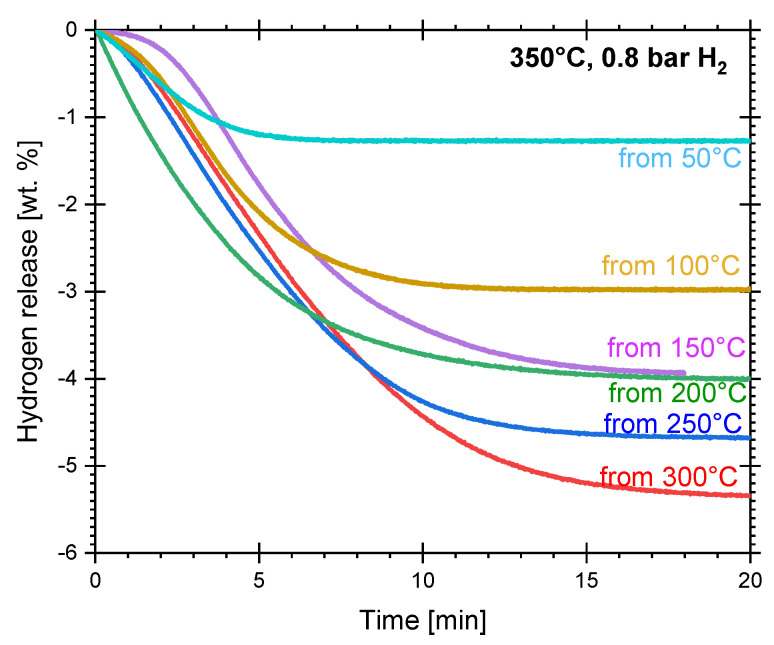
Isothermal dehydriding of cryogenically prepared Mg-15wt.% VCl_3_, previously hydrided at the indicated temperature. All dehydrogenations at 350 °C and 0.8 bar.

Figure 3 presents the isothermal dehydriding curves (350 °C and 0.8 bar absolute pressure). In this case, after hydriding, the pressure was reduced to 0.8 bar (equalizing to atmospheric pressure) and then the samples were quickly heated to 350 °C with the isolation valve closed. Once under isothermal conditions, the isolation valve was opened. Dehydriding reactions in such conditions of temperature and pressure are finished essentially in 20 min. Application of the kinetic models proposed in [42] gave the best fit with the two-dimensional JMA model (Equation (2), with n = 2). This indicates that the nucleation of the new phase begins randomly in the bulk and at the surface of particles. An example of the fitting process parameters is presented in Figure S1 of the supplementary file. Equation (2), with n = 2, best fits the data in the 1–10 min period [42,44]. The kinetic constants are presented in Table 2, but on average, k = 2.88 × 10^−3^ s^−1^.

**Figure 4:**
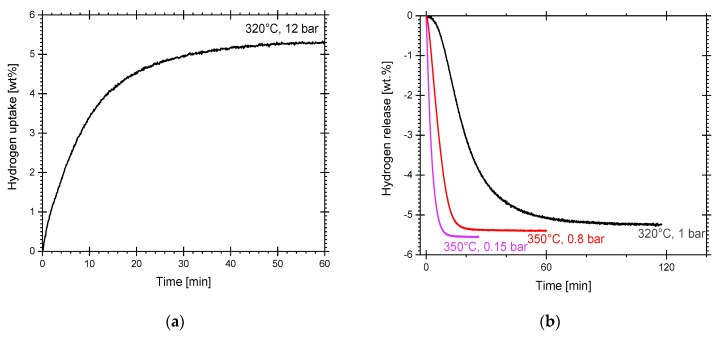
Hydriding/dehydriding cycles of cryogenically prepared Mg-15wt.% VCl_3_. (**a**) Isothermal hydriding at 320 °C/12 bar. (**b**) Isothermal dehydriding at 320 °C/1 bar. For comparison, isothermal dehydriding at 350 °C/0.15 bar and 350 °C/0.8 bar (red line of Figure 3) were included.

An additional isothermal hydriding/dehydriding cycle at 320 °C, 12 bar and 1 bar, respectively, is presented in Figure 4a,b. The isothermal cycle in that particular condition was performed to compare to a published report of Mg-5wt.% VCl_3_ milled in hydrogen [29]. The work in [29] is the closest report to our work due to the use of Mg as a precursor [29]. The reversible hydrogen storage level was 5.2 wt.% in the isothermal cycle of Figure 4a. Hydrogen storage level at 12 bar is quite similar to the isothermal experiment at 300 °C/26 bar presented above, but lower than at 350 °C/26 bar. Dehydriding reaction is presented in Figure 4b. For comparison, isothermal dehydriding at 350 °C/0.8 bar (red line of Figure 3) and at 350 °C/0.15 bar (from another previous hydriding at 350 °C/12 bar, light-pink curves) are included. The curves indicate the dependency on pressure and temperature of dehydriding. As expected, at lower pressure the dehydriding reactions are quicker. Furthermore, 0.15 bar absolute pressure, in practical terms, requires operation under a certain vacuum level. Still, many reported works utilize such low pressures, for example in [15,30,45]. Dehydriding at 320 °C/1 bar constitutes a bit more practical operation conditions. Under such conditions, dehydriding takes about 70 min to complete.

**Figure 5:**
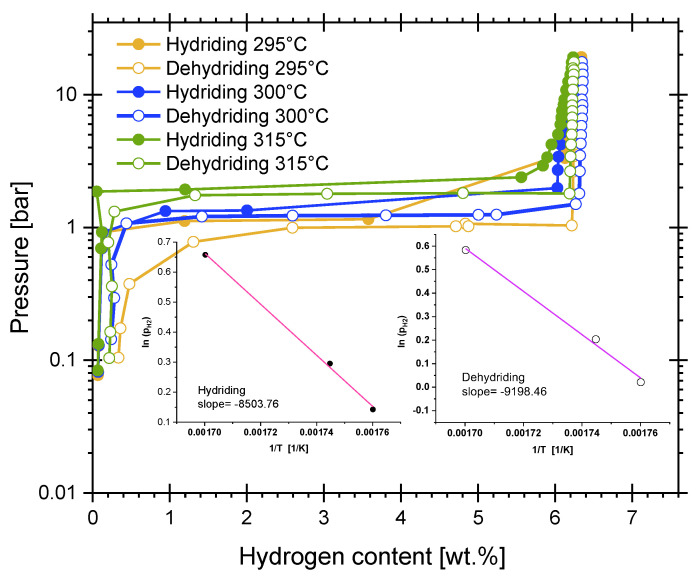
PCI experiments at different temperatures of cryogenically prepared Mg-15wt.% VCl_3_ (shown in absolute pressure). Insets: hydriding and dehydriding equilibrium pressures versus 1/T for estimation of the hydriding and dehydriding enthalpies and entropies.

Figure 5 presents the results of PCI experiments in a relatively narrow temperature range (295–315 °C). At 300 °C, the calculated equilibrium pressure (Equation (5)) of pure Mg ranges between 1.81 bar (for ΔH^0^_f_ (MgH_2_) = −74.6 kJ/mol and ΔS^0^ (H_2_) = 135 J/mol*K [46]) and 0.71 bar (for ΔH^0^_f_ (MgH_2_) = −76.15 kJ/mol and ΔS^0^ (H_2_) = 130 J/mol*K [47]).

(5) $$ln\left(\frac{p_{eq}}{p_{eq}^{0}}\right)=-\frac{{∆H}_{f\left(MgH2\right)}^{0}}{RT}+\frac{{∆S}_{\left(H2\right)}^{0}}{T}$$

The experimental hydriding equilibrium pressure of Mg-15wt.% VCl_3_ was located at 1.34 bar at 300 °C. Meanwhile, the dehydriding equilibrium pressure at the same temperature was 1.22 bar. For the rest of the hydriding and dehydriding reactions, the values are 1.93 bar and 1.79 at 315 °C; and 1.15 bar and 1.02 bar at 295 °C; respectively. These values indicate a relatively small hysteresis in the hydriding/dehydriding reactions of cryogenically milled Mg-15wt.%VCl_3_. The insets of Figure 5 are the plots of the *ln*(*p_eq_*) versus the inverse of temperature for the hydriding and dehydriding reactions. Hydriding and dehydriding enthalpies and entropies were obtained from the slope and the intercept of the linear fit of experimental equilibrium data and Equation (5). The values are −70.69 ± 3.08 kJ/mol H_2_ and −76.47 ± 5.71 kJ/mol H_2_, for hydriding and dehydriding enthalpies, respectively. Meanwhile, the hydriding and dehydriding entropies were estimated as 125.7 ± 5.3 J/mol*K and 134.9 ± 9.9 J/mol*K, respectively. However, the calculation of hydriding reaction enthalpy can be influenced by the fact that the hydriding PCI curves were constructed with few data points. The calculation from the dehydriding reaction is more reliable. Still, the calculated hydriding equilibrium pressures correlate well with the expected Mg/MgH_2_ equilibrium. The equilibrium pressures and the enthalpy values indicate a slight effect of the addition of VCl_3_ on the fundamental thermodynamics of the Mg/MgH_2_ system. Reported values of equilibrium pressures of MgH_2_-VCl_3_ are a bit higher than those encountered for our Mg-15wt.%VCl_3_ material: at 300 °C, da Conceicão et al. reported PCI plots of MgH_2_-7wt.% VCl_3_, where a hydriding equilibrium pressure of about 1.8 bar can be read [30]. For its part, Liang et al. reported a hydriding equilibrium pressure of about 1.9 bar for MgH_2_-5at.% V at 300 °C [45]. Malke et al., for a series of mixtures of MgH_2_ with transition metal halides (7 wt.%, mainly fluorides), reported a hydriding equilibrium pressure at about 1.5–1.6 bar at 300 °C [16].

**Figure 6:**
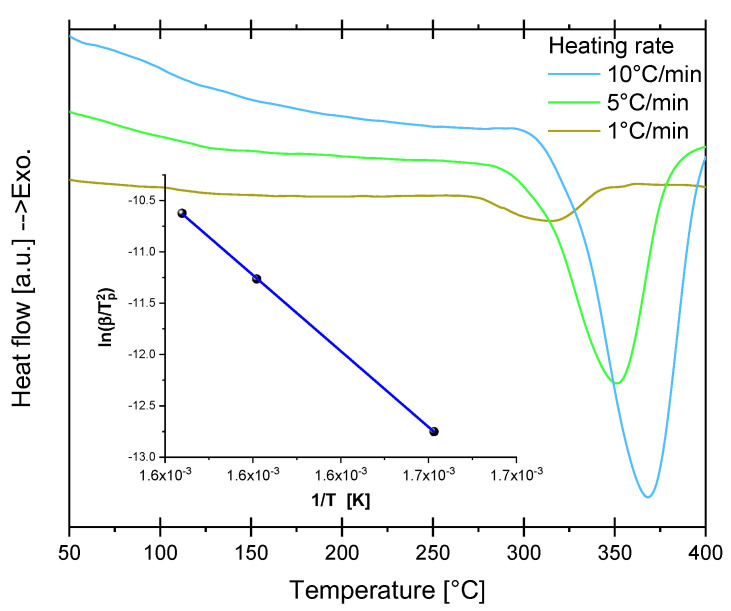
DSC trace of the dehydriding reaction of cryogenically prepared Mg-15wt.% VCl_3_ hydrided at 350 °C/26 bar. Inset: Application of the Kissinger method for estimation of the dehydriding activation energy.

Dehydriding was also studied by DSC (Figure 6). DSC experiments confirm the high temperature required for dehydriding the cryogenically prepared Mg-15wt.% VCl_3_. At a 1 °C/min heating rate, the dehydriding peak temperature was 314 °C with an onset at about 280 °C. Application of the Kissinger method [48,49] (inset of Figure 6, Equation (6)) allows the obtention of the activation energy.

(6) $$ln\left(\frac{\beta }{T_{p}^{2}}\right)=\ln\left(\frac{A*R}{E_{a}}\right)-\frac{E_{a}}{RT_{p}}$$

In Equation (6) β is the heating rate, *T_p_* is the dehydriding peak temperature, *A* is a pre-exponential factor and *E_a_* is the activation energy. A dehydriding activation energy of 123.11 ± 0.61 kJ/mol H_2_ was obtained from the slope of the linear fitting of the inset of Figure 6. This value is similar to other values of activation energies reported for MgH_2_ mixtures (more details in the Discussion section). This indicates that the added VCl_3_ has a small effect on the dehydriding reaction of hydrided Mg-15wt.% VCl_3_.

### 3.2. Hydriding and Dehydriding Reactions of Pure, Cryogenically Milled Mg and Mg-15wt.% VCl_3_-RT

**Figure 7:**
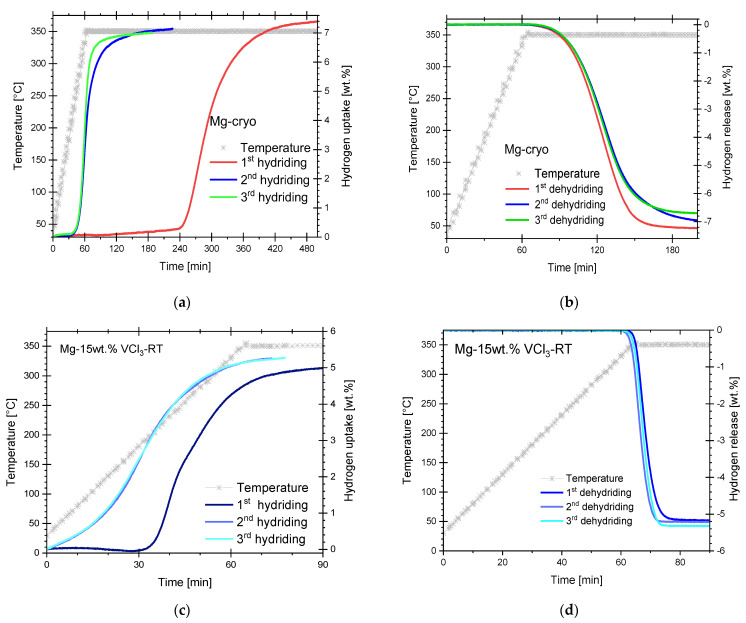
(**a**) Hydriding/(**b**) dehydriding cycles of cryogenically milled Mg. (**c**) Hydriding/(**d**) dehydriding cycles of Mg-15wt.%-RT (milled at room temperature). Hydriding at 26 bar hydrogen pressure. Dehydriding at 0.8 bar hydrogen pressure.

From published papers and our own experience, milling pure Mg without additives (including H_2_ in reactive milling) at room temperature is dominated by cold-welding [50,51,52]. Proper cryomilling conditions can reduce cold welding. Additionally, it is well established that achieving hydrogen uptake from pure Mg in moderate conditions of pressure is difficult [53]. Figure 7a,b presents a few hydriding/dehydriding cycles of pure Mg milled in cryogenic conditions. The first hydriding reaction was marked by a prolonged incubation period of almost 6 h, followed by slow hydriding kinetics, Figure 7a. This response is very much in common with other Mg/MgH_2_ reported samples prepared by mechanical milling at room temperature [51,52]. However, the kinetics of the second and third hydriding reactions were quite improved. A complement to the activation process described in the experimental section is hydriding/dehydriding cycling, as observed in this round of experiments. The hydrogen uptake levels of the second and third hydriding reactions are comparable to the first hydriding of Mg milled for 46 h at cryogenic conditions as reported elsewhere [54]. The second and third hydriding presented an onset temperature of 215 °C and 203 °C, respectively. However, upon cycling, a small reduction of the hydriding level can be observed (from 7.4 to 7.0 wt.%); this is due to the incomplete dehydriding reaction, Figure 7b. Interestingly, dehydriding reactions are quite similar, i.e., no noticeable effects of activation and cycling were observed on hydriding reactions. Furthermore, 0.1 wt.% of hydrogen release at 0.8 bar hydrogen pressure occurred after reaching 350 °C, after 77 min in the first dehydriding.

Figure 7c,d presents a mixture of Mg and VCl_3_ milled at the same conditions of materials proportion, time, pauses, agitation, etc., as the cryogenic material but a room temperature; i.e., the Mg-15wt.% VCl_3_-RT. This material required activation and cycling to improve hydriding kinetics and hydrogen storage level. A clear improvement of the hydriding kinetics was observed in the second cycle. However, the hydrogen uptake was only about 5.2–5.4 wt.%. This value is lower than the values obtained for the cryogenic mixture of Mg and VCl_3_. Additionally, the VCl_3_ and room-temperature ball milling have a small effect on the dehydriding reaction. The results presented in this section illustrate the beneficial effects of cryogenic milling on hydriding properties of Mg. In addition, they help to understand the effect of VCl_3_ on cryogenically milled Mg: VCl_3_ reduced incubation times and improved the kinetics of both hydriding and dehydriding reactions.

### 3.3. Characterization of As-Milled, Hydrided, and Dehydrided Materials

**Figure 8:**
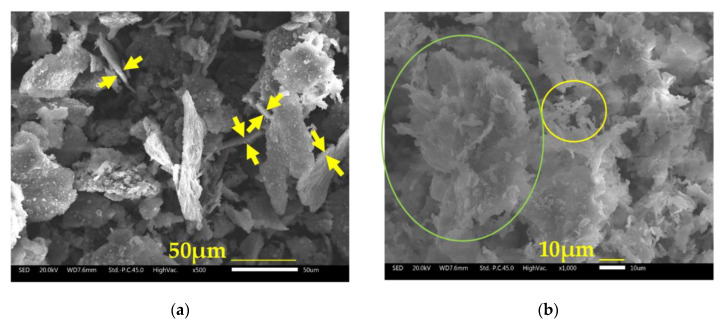
(**a**) SEM image of as cryogenically milled Mg-15wt.% VCl_3_. (**b**) SEM image of hydrided (at 350 °C and 26 bar) Mg-15wt.% VCl_3_ material. Green oval: flake. Yellow oval: dendritic growth.

Mg is a ductile material, difficult to mill without grinding additives [51,55]. However, cryogenic temperatures induces less ductility in materials and, in principle, fracturing may dominate over cold welding [35]. Figure 8 presents SEM images of the as-milled and hydrided Mg-15wt.% VCl_3_. As mentioned in the experimental section, the starting size of the purchased Mg powders was quoted as −325 mesh (<44 µm); however, the initial material has a maximum particle size of about 200 µm and is of irregular shape (Figure S2, Supplementary File). In the cryogenically milled Mg-15wt.% VCl_3_ material, Figure 8a, a flattening of the particles occurred during milling; the final morphology after 1 h of cryomilling is similar to “corn flakes”. This indicates that Mg is a ductile material even at cryogenic temperatures. The length of most as-milled particles is between 50 and 100 µm, but some of them extended up to 200 µm (Figure S3, Supplementary File). The arrows in Figure 8a indicate some of the measurement sites for the thickness of the flakes. Tilted particles were not taken into account for the estimation of thickness. We estimated the thickness of the flakes between 2.5 and 5.0 µm. In comparison, the original powders (Figure S2, Supplementary File) had an initial thickness of roughly 15–30 µm. The SEM images presented in Figure 8 and the Supplementary File demonstrate a reduction in particle thickness that allows for a shorter diffusion pathway. Elemental mapping of Mg-15wt.% VCl_3_ (Figure S4, Supplementary File) demonstrated a good dispersion of VCl_3_ onto Mg particles. Additionally, SEM images in Figure 8 reveal surface charging, which suggests the presence of non-conductive phases such as MgO or Mg(OH)_2_. Non-conductive phases are usually more brittle and therefore easier to downsize by cryogenic ball-milling. The presence of MgO or Mg(OH)_2_ can decrease the hydrogen uptake and can explain part of the “missing” 0.46 wt.% in the achieved hydrogen storage capacity (6 wt.%, Figure 1) versus the maximum expected value (6.46 wt.%).

The Mg powders milled in cryogenic conditions produced a mixture of globular particles with some flakes of about 100 µm (Figure S5, Supplementary File). Therefore, VCl_3_ acts both as a grinding additive and as a catalyst for the hydriding reaction of Mg. Other transition metal halides, NbF_3_ and FeF_3_ (2 mol%), were recognized as efficient grinding additives (3 h cryomilling) when added to pure MgH_2_ [10]. From our experience, the micro-flaked morphology of as-milled Mg-15wt.% VCl_3_ is quite peculiar. Comparison with other Mg-based materials is difficult because of the different precursors (MgH_2_ vs. Mg) and milling conditions (room temperature vs. cryogenic) or the presence of other co-catalysts. Still, upon room-temperature milling, the partial production of Mg flakes was reported under particular milling conditions [55]; otherwise, other morphologies were obtained. For example, MgH_2_-VCl_3_ processed by room-temperature milling produced globular particles with high surface rugosity [26]. Other reports of room-temperature milled MgH_2_-VCl_3_, unfortunately, did not include SEM images. SEM images of cryogenically prepared Mg-mixtures also are scarce. Floriano et al., included SEM images of MgH_2_ milled with iron or niobium compounds in cryogenic conditions, and showed the formation of granular particles with bimodal size of dispersions, 10–25 µm and 0.25–0.3 µm [10]. Another reference to cryomilling of Mg involved a Mg-Fe mixture, where the resulting quasi-spherical particles were of smaller size than their counterparts produced by room-temperature planetary milling [35]. Comparing these reports and the results presented here, we conclude that the milling time, the starting material Mg (vs. MgH_2_), the use of cryogenic milling, and the VCl_3_ addition played an important role in the size and morphology of the products.

Figure 8b presents an SEM image of hydrided Mg-15wt.% VCl_3_. The hydrided material corresponds to the fourth hydriding reaction of Figure 1a. Changes in morphology upon hydriding/dehydriding reactions are complex. On one side, fragmentation of the original Mg particles occurs due to the expansion/contraction of the crystalline cell during hydriding/dehydriding cycling. On the other side, agglomeration of the smaller particles can be observed in Figure S6, Supplementary File. Agglomeration makes it difficult to obtain a precise estimation of individual particle sizes. Still, the shape and size of some of the original Mg-15wt.% VCl_3_ particles seem to remain in the cycled flakes (green oval in Figure 8b). The formation of dendritic material at the surface of the flakes (yellow oval in Figure 8b) during cycling is interesting. Elemental mapping of cycled material is presented in Figure S7, Supplementary File; a good dispersion of VCl_3_ onto Mg is observed.

**Figure 9:**
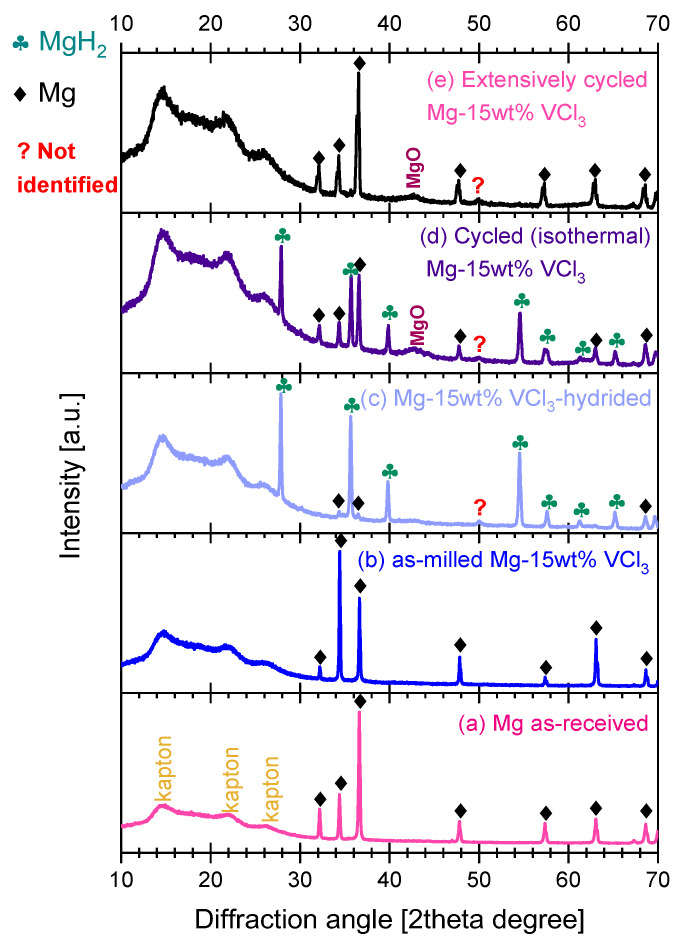
X-ray diffraction patterns of (**a**) as-received Mg, (**b**) cryogenically milled Mg-15wt.% VCl_3_, (**c**) material hydrided at 350 °C/26 bar of Figure 1, (**d**) material from the hydriding/dehydriding isothermal cycling of Figure 2 and Figure 3; (**e**) from extensive PCI cycling.

Figure 9 presents the X-ray diffraction characterization at several stages (as-milled, hydrided, dehydrided) of Mg-15wt.% VCl_3_. The first observations are the presence of the peaks of the Kapton film between 10 and 25° and the strong background common to all samples, also due to the Kapton. The X-ray diffraction pattern of as-received Mg (Figure 9a) was included for comparison. The peaks of Kapton film can be used as a visual reference for the changes in relative intensities of the diffraction peaks. The first characteristic to mention of the as-milled Mg-15wt.% VCl_3_ (Figure 9b) is the change in relative intensities of some peaks. In the as-milled mixture, the Mg (100), (101), and (110) *hkl* peaks (32.18°, 36.61°, and 57.37°, respectively) are less intense than expected for a powder without texture (ICSD-642651). Meanwhile, Mg (002), at 34.39°, presented higher than expected intensity, as frequently reported in plastically deformed Mg alloys [55]. These characteristics are not observed in pure, cryogenically milled Mg (Figure S8, Supplementary File). In Mg-15wt.% VCl_3_, the texture can be attributed to the flat shape of the particles that induce a preferential orientation during the compaction of the powders for XRD data collection. Additionally, the VCl_3_ peaks are missing (see Figure S9, Supplementary File, for XRD of the original VCl_3_ powders). In our experience, VCl_3_ easily amorphizes by ball-milling, even in cryogenic mode, and this would explain the lack of the corresponding XRD peaks. The cryogenically milled Mg-15wt.% VCl_3_ is a fine mixture of Mg microflakes and non-crystalline VCl_3_.

The X-ray diffraction pattern of hydrided Mg-15wt.% VCl_3_ material is presented in Figure 9c. This material corresponds to the fourth hydriding step of Figure 1a. The hydriding conditions lead to an almost complete transformation to MgH_2_, with a minor presence of unreacted Mg. Additionally, the emergence of an unidentified minor peak was observed. We revised the possible match of that peak to the expected XRD patterns of MgCl_2_, VCl_2_, or V-hydrides, without positive identification. Figure 9d presents the XRD patterns of cycled materials corresponding to the hydriding/dehydriding isothermal cycling in Figure 2 and Figure 3. The material after isothermal cycling was not forced to dehydride before XRD data collection; this is a partially dehydrided material. First to mention is that the Mg texture found in the XRD pattern after cryogenic ball-milling disappeared after cycling, with the peak intensities being now as expected from randomly oriented Mg particles. Additionally, the relative intensity of Mg peaks versus Kapton peaks diminished after cycling (more clearly observable in the extensively cycled material, Figure 9e). A slight broadening of the diffraction peaks was also registered. This suggests the reduction of crystallite size by the effect of the fracturing of particles/crystals during cycling. Next to mention is the presence of a broad peak at about 43° in 2theta, which is consistent with the formation of some amount of MgO by the effect of heating in the presence of impurities in the hydrogen gas, and/or the decomposition of superficial Mg(OH)_2_. Figure 9e presents the XRD pattern of an extensively PCI cycled material (additionally to the number of PCI cycles presented in Figure 5). The material of Figure 9e confirmed the reduction of the peak intensity and a slight increase in width of Mg diffraction peaks compared to the as-milled material.

## 4. Discussion

### 4.1. Remarks on Cryogenic Milling and Amount of Added VCl_3_

Cryogenic ball milling produced interesting hydriding behavior on pure Mg. The activation towards MgH_2_ formation was relatively easy and comparable with other materials produced with long milling times in cryogenic conditions [54]. For a given mill and ball size, factors such as the ball-to-powder ratio, the agitation rate of the vial (25 Hz), and milling/pause time play an important role in the energy transferred to the milled powders. The addition of VCl_3_ facilitated the activation, drastically reducing the incubation time of hydriding and dehydriding reactions, and allowing hydriding at low temperatures. However, the “price” to pay is the reduction of hydrogen storage capacity. Thus, further optimization of the amount of VCl_3_ is needed. Up to now, the published reports do not show a clear improvement with increasing additions of VCl_3_ (from 5 wt.% to 23.9 wt.%, i.e., up to 5 mol%, Table 3) [25,26,29,30,31]. Table 3 collects the reported results on mixtures of Mg or MgH_2_ with VCl_3_.

### 4.2. Other Reported Mg/MgH_2_-VCl_3_ Mixtures

A direct comparison of the kinetics and the amount of hydrogen storage of our Mg-15wt.% VCl_3_ material versus published reports is difficult. Different conditions regarding the precursors (Mg vs. MgH_2_), amount of VCl_3_, sample preparation, and testing conditions can influence the results. Even more, with the same material but different characterization techniques, different results were reported (Table 3, ref. [25]).

Based on our experience, mixtures starting with Mg can present a more challenging activation or reduced hydriding/dehydriding kinetics as compared to mixtures starting from MgH_2_. The work of Song et al. [29] is similar to ours due to their use of Mg as the precursor. The differences between our work and Song et al’s. [29] is in the use of cryomilling versus reactive milling in hydrogen and the amount of VCl_3_ addition. The last factor affected the maximum theoretical uptake: 6.46 wt.% here and 7.22 wt.% in ref. [29]. We use in Table 3 the same time-markers as Song et al. [29], i.e., hydriding and dehydriding levels at 2.5 and 60 min. In both works, the hydriding and dehydriding levels are more or less comparable, considering the corresponding experimental conditions of P and T. Hydriding kinetics is faster in our cryogenically prepared Mg-15wt.% VCl_3_ material. The dehydriding reaction started faster in the work of Song et al. [29], but by the end of the experiment (60 min), our cryogenically milled material desorbed more hydrogen. Due to safety concerns in reactive milling in hydrogen atmosphere, we recommend using instead cryogenic ball-milling of Mg. Among the materials that used MgH_2_ as MgH_2_-5 wt.% VCl_3_ precursor stands out as produced by Kumar et al. [26]. However, no detailed data on dehydriding kinetics were presented by these authors. Finally, Table 3 presents three outstanding reported materials: MgH_2_/NiCl_3_, MgH_2_/TiCl_3,_ and MgH_2_/NbCl_5_ to have an idea of the performance of other transition metal chlorides used as accelerators of hydriding/dehydring of MgH_2_. A direct comparison is not an easy task due to the differences in precursor, milling conditions, and quantity of the transition metal chloride added. Optimization of the milling process and quantity of VCl_3_ must be performed in further work. Still, the use of VCl_3_ as a hydriding/dehydriding accelerator can be another viable option for catalyzing the hydriding/dehydriding reactions of Mg/MgH_2_.

### 4.3. Hydriding Activation Energy

Table 4 compares data on hydriding activation energy from pure Mg and our Mg-15wt.% VCl_3_ material. An important reduction in the hydriding activation energy has occurred for the cryogenically milled Mg-15wt.% VCl_3_. As mentioned before, the cryogenically milled Mg-15wt.% VCl_3_ exhibits fast kinetics in the different tested conditions of pressure and temperature. Fast kinetics is a good characteristic of any Mg-based material [59]. In our case, fast kinetics is the result of the favorable activation energy.

### 4.4. Dehydriding Activation Energy

Hydriding activation energy data (Table 4) are scarcer than dehydriding activation energy data (Table 5) because MgH_2_ is more frequently used as a starting material than Mg, and because of the interest in finding softer conditions for hydrogen release. However, reported values of activation energy for the dehydriding reaction are very dispersed, perhaps because of the different conditions of materials processing (materials history), and experimental conditions of data collection for activation energy determination. In Table 5 we collected relevant data reported on the dehydriding reaction in MgH_2_-based materials. The dehydriding activation energy of the cryogenically milled and hydrided Mg-15wt.% VCl_3_ is lower than that of pure MgH_2_. The dehydriding activation energy reported here is similar to that of MgH_2_ containing other transition metal chlorides (including a MgH_2_ -VCl_3_ mixture from Ref [26], NiCl_2_, TiCl_3_ and NbCl_5_). In addition it is higher than other V or VCl_3_-added MgH_2_ materials [25,30,45]. Among the data collected in Table 5, there is no clear trend between the VCl_3_ addition level, particle size, milling conditions, and activation energy.

### 4.5. Reason for Improved Hydriding Kinetics

In an interesting report, Ma et al. proposed the occurrence of Ti-F-Mg interactions in TiF_3_-doped MgH_2_ as the cause for improved kinetics of MgH_2_ [56]. However, a similarly produced TiCl_3_-doped MgH_2_ did not show such kind of interactions (Ti-Cl-Mg) [56]. However, Mg and Ti interaction with Cl can be expected because the TiCl_3_-doped MgH_2_ also showed an improvement compared to Mg alone. An example of another interaction between a catalyst and Mg is the efficient Nb_2_O_5_ catalyst interacting with Mg to form MgNb_x_O_y_ species [59,64]. In the present work, we proposed a V-Cl-Mg interaction as being responsible for the improvement in hydriding/dehydriding kinetics in our Mg-15wt.% VCl_3_ material. We are performing further work to unravel properly the kind of interactions on the system Mg-15wt.% VCl_3_, but a strong dependency on the oxidation state of V can be anticipated.

On the other hand, the reaction between MgH_2_ and the additives has been frequently reported. Kumar et al. indicated the thermodynamic feasibility of the reaction between MgH_2_ and VCl_3_ even at room temperature [26]:

VCl_3_ + 1.5MgH_2_ → 1.5MgCl_2_ + V + 1.5 H_2_, ΔG_r_ = −322.24 kJ/mol (7)

However, despite their thermodynamic feasibility, neither MgCl_2_ nor V was detected by XRD. Thus, the occurrence of Equation (7) in our system is limited. We also discard the reaction between Mg and V (Equation (8)) during cryomilling or heating, because of the lack of VCl_2_ detection by XRD.

VCl_3_ + Mg → MgCl_2_ + 0.5VCl_2_ + 0.5V, (8)

At near room temperatures and moderate pressures, V can form several hydrides (V_2_H, VH, VH_2_, and non-stoichiometric hydrides) [65,66,67]. The equilibrium pressure of VH_2_ formation is reported to be 3–5 bar at low temperatures (room-temperature to ~100 °C) [65,66,67]. Additionally, the equilibrium pressure of V-hydrides can be modified by alloying [67]. In the present work, the formation of V-hydrides was not confirmed by XRD. Additionally, no indications of another equilibrium plateau, besides the one of Mg/MgH_2_, are observable in the PCI curves. The occurrence of V-hydrides would be linked to the occurrence of reactions (7) and (8).

## 5. Conclusions

Cryogenically prepared mixtures of Mg-15wt.% VCl_3_ were tested for hydrogen storage purposes. Cryogenic milling and the use of VCl_3_ as an additive produced an uncommon morphology of the as-milled powders. The milled material is a fine mixture of components. The Mg-15wt.% VCl_3_ demonstrated easy hydrogen uptake even at low temperatures because of a reduction of the activation energy of the hydriding reaction, as compared to reported data on pure Mg. The activation energy of the dehydriding reaction also diminished as compared to pure Mg. However, the complete dehydriding reaction occurred only at high temperatures (300–350 °C) at 1 bar hydrogen pressure. Kinetics models point to the diffusion of hydrogen atoms as the main factor affecting the hydriding reaction. The cryogenic ball-milling opens the way for a preparation procedure that can be applied to other combinations of Mg and transition metal salts.

## Figures and Tables

**Table 1:** Rate constant at different temperatures for the hydriding reaction of cryogenically prepared Mg-15wt.% VCl_3_.

Temperature [°C]	Time Interval [min]	k [s^−1^]
50	0–60	3.05 × 10^−5^
100	0–19	6.06 × 10^−5^
150	0–12	7.58 × 10^−5^
200	0–0.1	1.51 × 10^−3^
250	0–0.3	3.73 × 10^−3^
300	0–0.5	4.60 × 10^−3^

**Table 2:** The rate constants for the dehydriding reaction of cryogenically prepared Mg-15wt.% VCl_3_ at 350 °C and 0.8 bar absolute pressure.

Previous Hydriding Temperature [°C]	k [s^−1^] of Dehydriding at 350 °C, 0.8 Bar
50	4.98 × 10^−3^
100	3.14 × 10^−3^
150	2.50 × 10^−3^
200	2.24 × 10^−3^
250	2.38 × 10^−3^
300	2.05 × 10^−3^

**Table 3:** Comparison of material composition, preparation, hydrogen uptake and release, and dehydriding temperature of reported Mg/MgH_2_-VCl_3_ and other mixtures.

Material, Theoretical Hydrogen Content [wt.%], and Highlights of Preparation	Ref.	Hydrogen Uptake [wt.%]	Hydrogen Release [wt.%]	Peak Dehydriding Temperature [°C] and Conditions
Mg-5 wt.% VCl_3_ 7.22 milled in hydrogen	[29]	3.37 wt.% at 2.5 min 6.36 wt.% at 60 min 320 °C, 12 bar, isothermic.	0.12 wt.% at 2.5 min 4.54 wt.% at 60 min 320 °C, 1 bar, isothermic.	Not reported
Mg-15 wt.% VCl_3_ 6.46 1 h cryomilling	This work	5.19 wt.% at 2.5 min 5.6 wt.% at 60 min 300 °C, 26 bar, isothermic.	0.95 wt.% at 2.5 min 5.34 wt.% at 20 min 350 °C, 0.8 bar, isothermic.	314 °C, 1 °C/min, DSC
		1.26 wt.% at 2.5 min 5.3 wt.% at 60 min 320 °C, 12 bar, isothermic.	0.03 wt.% at 2.5 min 5.07 wt.% at 60 min 320 °C, 1 bar, isothermic.	
MgH_2_-14 wt.% VCl_3_ 6.53 1 h milling in argon	[25]	Not reported	Not reported	~310 °C *, 5 °C/min, TPD
MgH_2_-7 wt.% VCl_3_ 7.06 1 h milling in argon	[25]	Not reported	Not reported	~305 °C *, 5 °C/min, TPD
				278 °C, 5 °C/min, DSC
MgH_2_-5 wt.% VCl_3_ 7.22 Planetary ball milling	[26]	~7.0 wt.% * 100 °C, 20 bar, 60 min, isothermic	~7.0 wt.% * TG-DSC No kinetics study	~275 °C, 1 °C/min, TG-DSC
MgH_2_-7 wt.%VCl_3_ 7.06 Pre-milled in H_2_ at 2 bar for 24 h. Then milled for 20 min with the additive.	[30]	~6.5 wt.% * 350 °C, 10 bar, 30 min Isothermic	~6.2 wt.% * 350 °C, 0.1 bar (vacuum), 14 min Isothermic	~375 °C DSC
MgH_2_-5mol% VCl_3_ (i.e., 23.9 wt.% VCl_3_) 5.78	[31]	wt.% not reported 270 and 320 °C, 100–150 bar In-situ SR-PXD	wt.% not reported 270 and 320 °C, dynamic vacuum In-situ SR-PXD	Not reported
MgH_2_-10 wt.% NiCl_2_ 2 h ball milled	[14]	~6.1 wt.% * 350 °C, 20 bar, 5 min, isothermic	~6.1 wt.% * 0.01 bar (vacuum), TPD	Not reported
MgH_2_-4mol% TiCl_3_ (i.e., 19.6 wt.% TiCl_3_) 10 h milling in argon	[56,57]	~5.1 wt.% * 300 °C, 20 bar, 1 min, isothermic	~3.8 wt.% * 280 °C, 0.1 bar (vacuum), 25 min, isothermic	Not reported
MgH_2_-5 wt.% NbCl_5_ milled in H_2_ at 2 bar for 24 h.	[58]	~6.4 wt.% * 350 °C, 10 bar, 15 min, isothermic	~6.4 wt.% * 350 °C, 0.1 bar (vacuum), 6 min, isothermic	~375 °C DSC

* As read from plots.

**Table 4:** The activation energy of the hydriding reaction.

Material	E_a_ [kJ/mol H_2_]	Reference
Mg	90 ± 10	[60]
	95–130	[61]
Mg-15wt.% VCl_3_	63.8 ± 5.6	This work

**Table 5:** Activation energy of dehydriding reaction.

Material	Relevant Conditions of Materials Preparation	E_a_ [kJ/mol H_2_]	Reference
MgH_2_	Not milled	240	[25]
MgH_2_	Commercial (not milled)	195.3 ± 10	[62]
MgH_2_	Not milled	156	[63]
MgH_2_	Particle size 45 µm	160 ± 10	[60,61]
MgH_2_	2 h ball milled	158.5	[14]
Mg-15wt.% VCl_3_	Cryogenic ball milling (2.5–5 µm thickness)	123.11 ± 0.61	This work
MgH_2_-5wt.% VCl_3_	Planetary ball milling, 2 h, particle size < 10 µm	122 ± 5	[26]
MgH_2_-10wt.% CoCl_2_	2 h ball milled	121.3	[14]
MgH_2_-10wt.% NiCl_2_	2 h ball milled	102.6	[14]
MgH_2_-7wt.% TiCl_3_	1 h milling in argon	97	[25]
MgH_2_-5wt.% NbCl_5_	24 h reactive-milling in 2 bar H_2_	98	[58]
MgH_2_-7wt.% VCl_3_	1 h milling in argon	96–97	[25]
MgH_2_-5 at.% V (9.2 wt.% V)	20 h milling, particle size < 5 µm	62	[45]
MgH_2_-7 wt.% VCl_3_	Pre-milled in H_2_ at 2 bar for 24 h. Then milled for 20 min with the additive.	47	[30]

## Data Availability

The data presented in this study are available on request from the corresponding author.

## Supplementary Materials

The following supporting information can be downloaded at: https://www.mdpi.com/article/10.3390/ma16062526/s1, Figure S1. Application of the kinetic models to the dehydriding reaction at 350 °C and 0.8 bar. Figure S2. Scanning electron micrograph (SEM) of the as-received Mg powders. Figure S3. Scanning electron micrograph (SEM) of cryogenically milled Mg-15wt.% VCl_3_. Figure S4. Elemental mapping of cryogenically milled Mg-15wt.% VCl_3_. Figure S5. Scanning electron micrographs (SEM) of cryogenically milled Mg. Figure S6. Scanning electron micrographs (SEM) of hydrided Mg-15wt.% VCl_3_, 4th cycle at 350 °C, 26 bar. Figure S7. Elemental mapping of cycled (PCI-cycled) Mg-15wt.% VCl_3_. Figure S8. X-ray diffraction of cryogenically milled pure Mg and hydrided material at 350 °C/26 bar. Figure S9. X-ray diffraction and SEM of as-received VCl_3_.
