# Supplementary material for: Fast Hydrogen Sorption Kinetics in Mg-VCl3 Produced by Cryogenic Ball-Milling
Source: Materials (Basel). 2023 Mar 22;16(6):2526. doi: 10.3390/ma16062526 (PMC10058655; doi:10.3390/ma16062526)

Fast hydrogen sorption kinetics in Mg-VCl3 produced by cryogenic ball-milling

Karina Suárez-Alcántara[1]*, Nadia Isabel Flores Jacobo[1], Mayara del Pilar Osorio-García[2], José Gerardo Cabañas-Moreno[2]

Figure S1. Application of the kinetic models to the dehydriding reaction at 350°C and 0.8 bar.

Equation	y = a + b*x					
Plot	Alpha	JMA 3D	JMA 2D	CV 3D	CV 2D	Cv-3D-Dif contro
Weight	No Weighting					
Intercept	0.04379 ± 0.004	0.40431 ± 0.002	0.16368 ± 0.001	-0.0685 ± 7.249	-0.05628 ± 8.685	-0.05863 ± 0.00
Slope	0.07362 ± 5.018	0.07835 ± 3.385	0.11446 ± 1.759	0.05029 ± 8.601	0.06202 ± 1.030	0.01968 ± 1.367
Residual Sum of S	2.17496	0.98983	0.26721	0.06389	0.09169	0.16149
Pearson's r	0.98235	0.99279	0.99908	0.99886	0.99892	0.98168
R-Square (COD)	0.96502	0.98564	0.99816	0.99772	0.99785	0.9637
Adj. R-Square	0.96497	0.98562	0.99816	0.99772	0.99785	0.96365

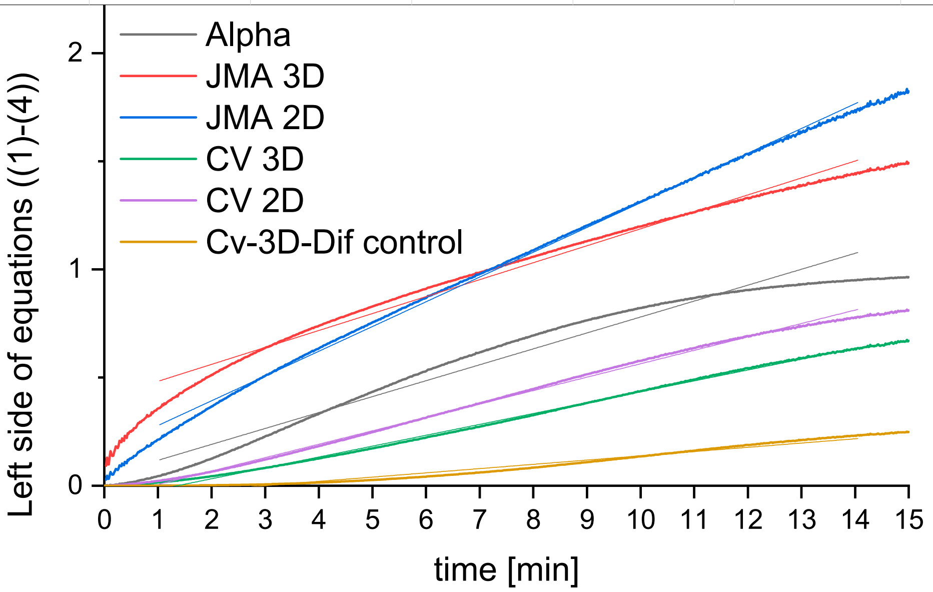

Figure S2. Scanning electron micrograph (SEM) of the as-received Mg powders.

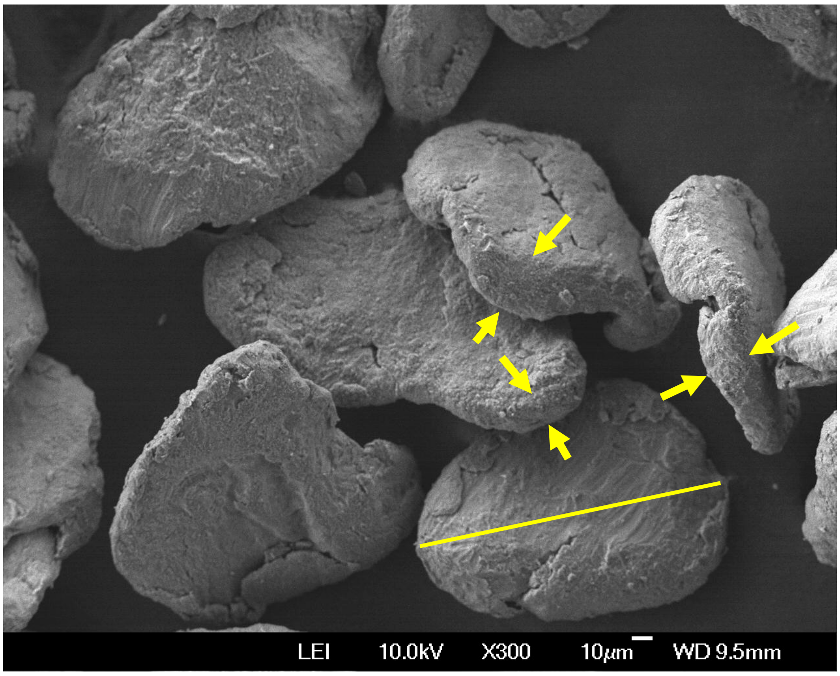

Figure S3. Scanning electron micrograph (SEM) of cryogenically milled Mg-15wt%VCl_3

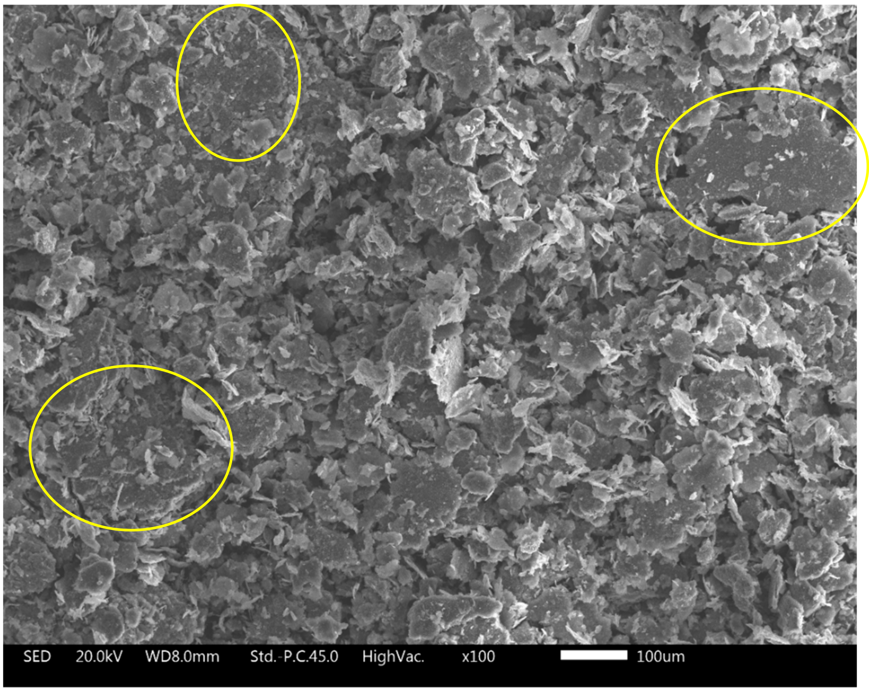

Figure S4. Elemental mapping of cryogenically milled Mg-15wt%VCl_3.

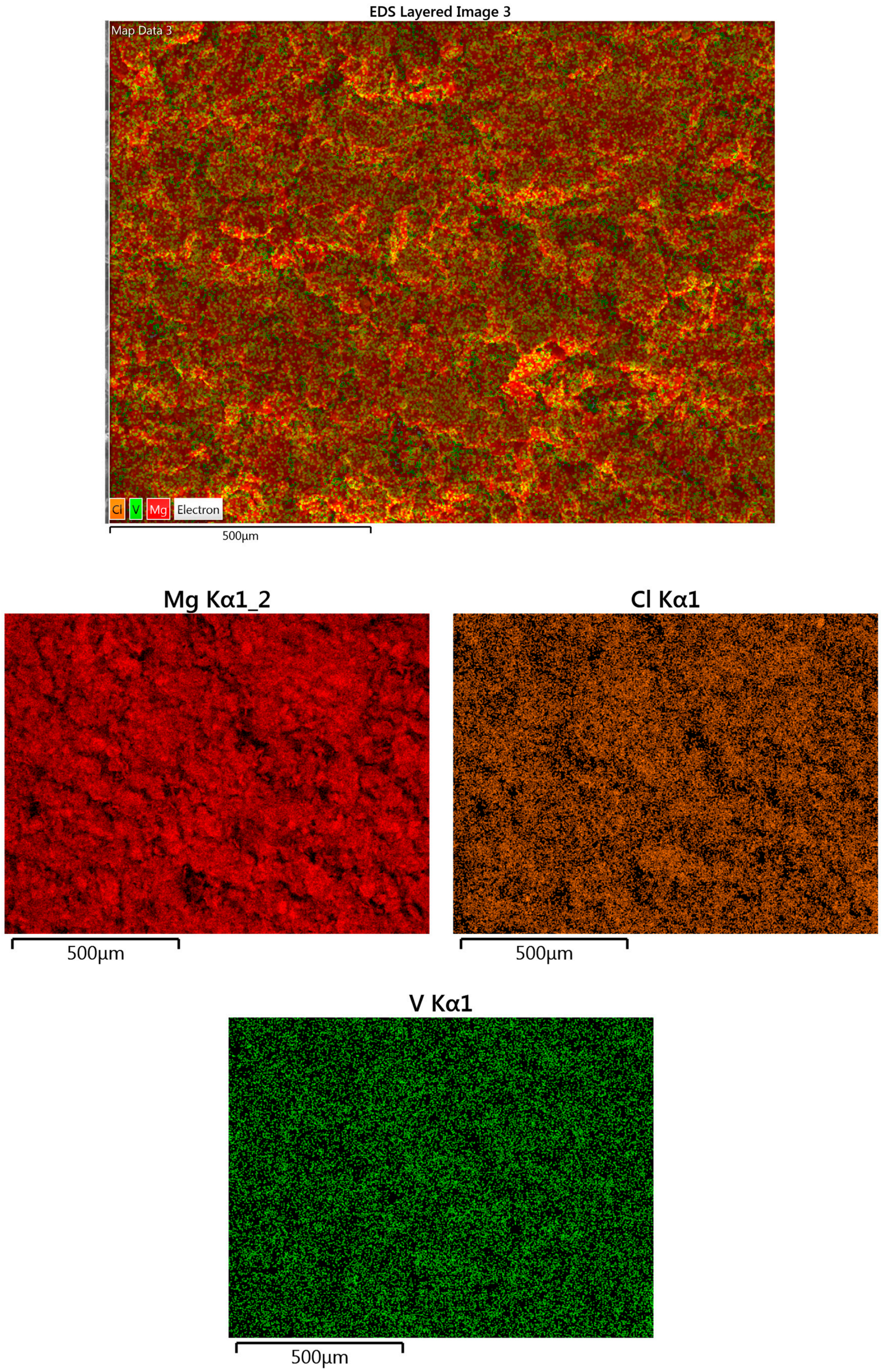

Figure S5. Scanning electron micrographs (SEM) of cryogenically milled Mg.

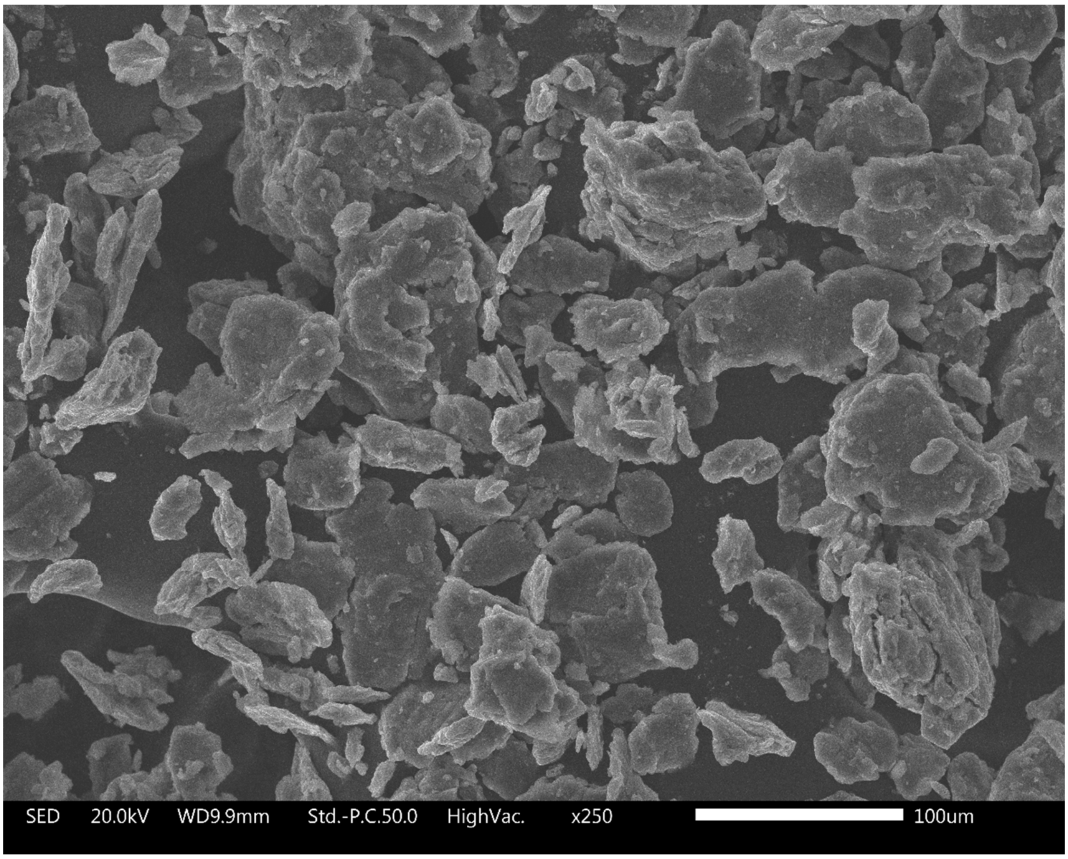

Figure S6. Scanning electron micrographs (SEM) of hydrided Mg-15wt%VCl_3, 4th cycle at 350°C, 26 bar.

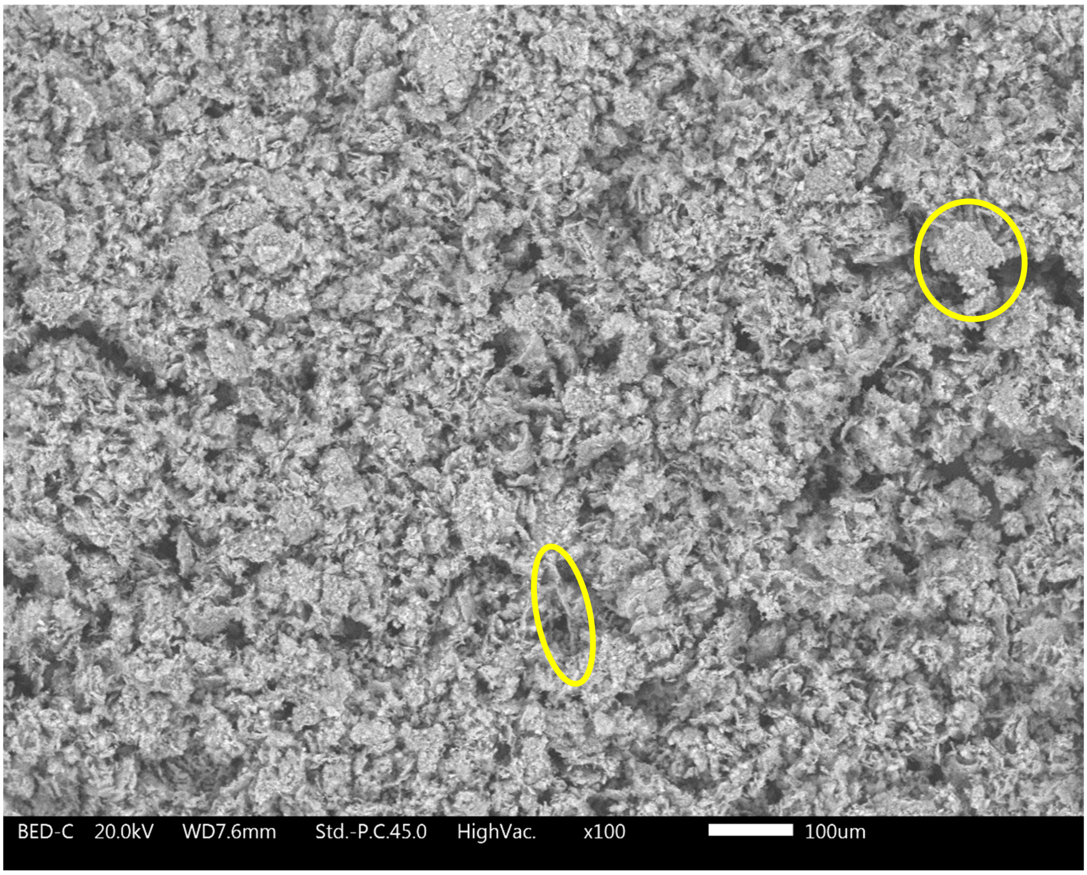

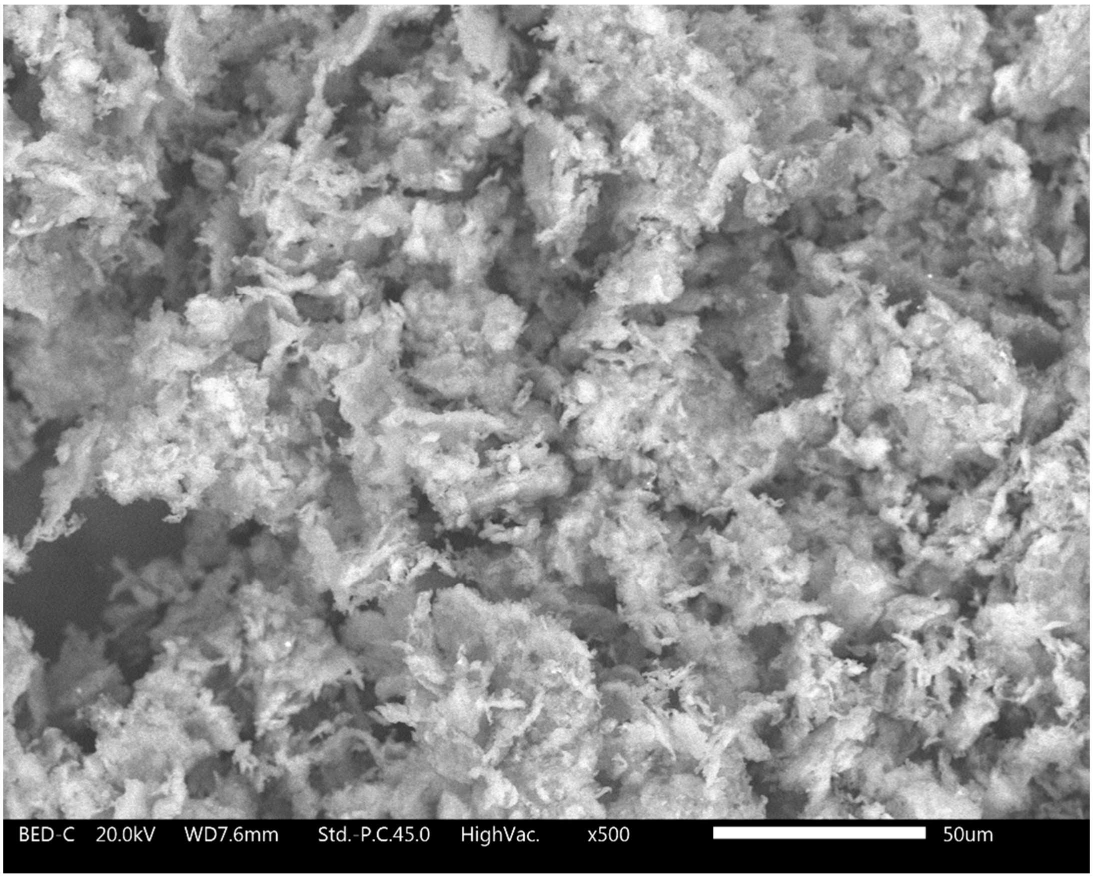

Figure S7. Elemental mapping of cycled (PCI-cycled) Mg-15wt%VCl_3.

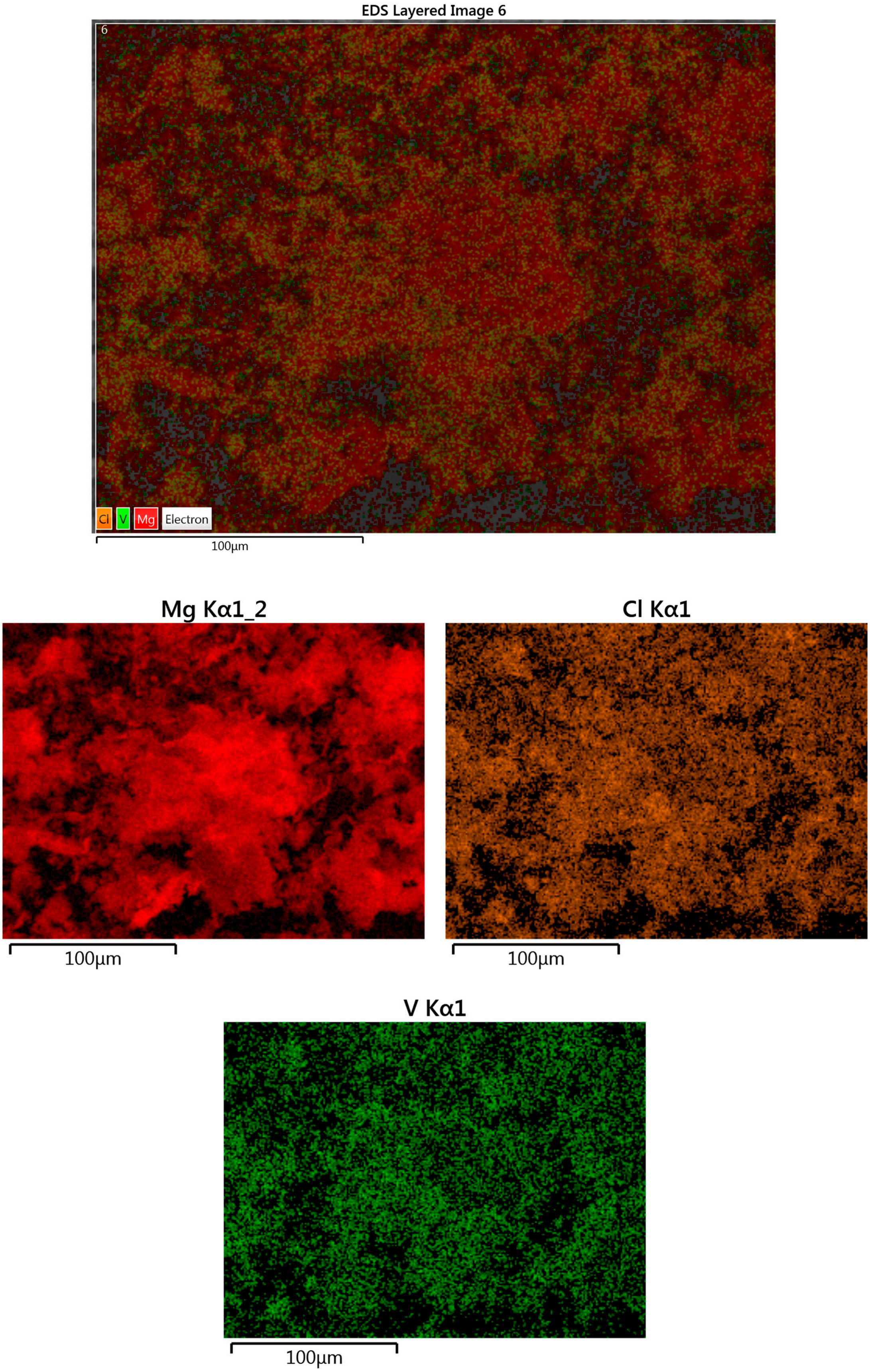

Figure S8. X-ray diffraction of cryogenically milled pure Mg and hydrided material at 350°C/ 26 bar.

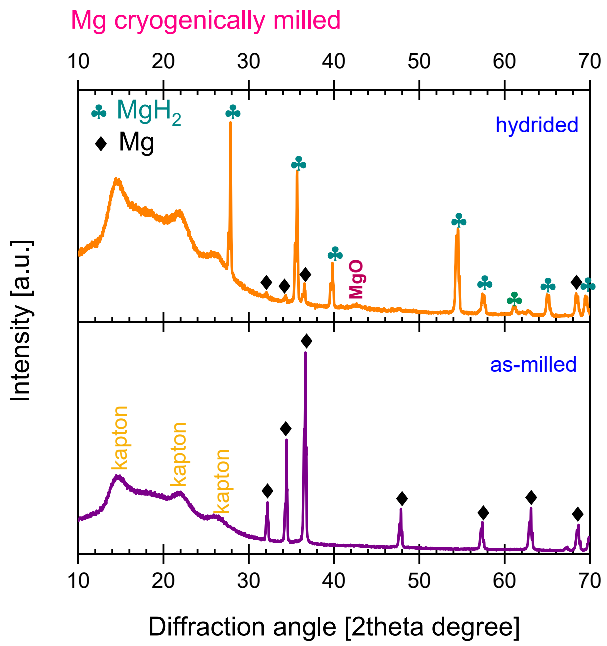

Figure S9. X-ray diffraction and SEM of as-received VCl_3.

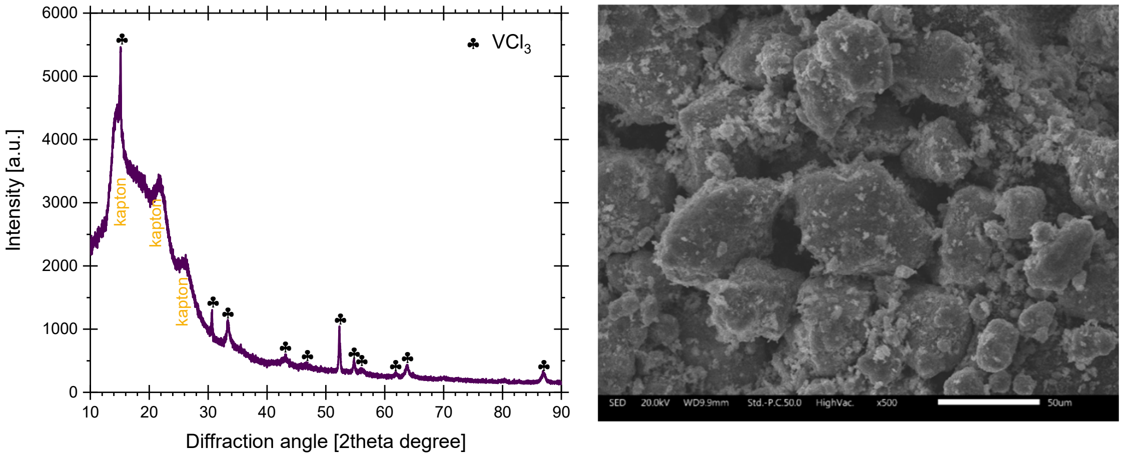

Supplement: Supplementary file 1 [file materials-16-02526-s001.zip › materials-2244718-supplementary.pdf]
